# Current and Future Use of Long Axial Field-of-View Positron Emission Tomography/Computed Tomography Scanners in Clinical Oncology

**DOI:** 10.3390/cancers15215173

**Published:** 2023-10-27

**Authors:** Mostafa Roya, Samaneh Mostafapour, Philipp Mohr, Laura Providência, Zekai Li, Johannes H. van Snick, Adrienne H. Brouwers, Walter Noordzij, Antoon T. M. Willemsen, Rudi A. J. O. Dierckx, Adriaan A. Lammertsma, Andor W. J. M. Glaudemans, Charalampos Tsoumpas, Riemer H. J. A. Slart, Joyce van Sluis

**Affiliations:** 1 Department of Nuclear Medicine and Molecular Imaging, Medical Imaging Center, University Medical Center Groningen, University of Groningen, P.O. Box 30001, 9700 RB Groningen, The Netherlands; s.mostafapour@umcg.nl (S.M.); p.mohr@umcg.nl (P.M.); l.lopes.goncalves.da.providencia@umcg.nl (L.P.); z.l.li@umcg.nl (Z.L.); j.h.van.snick@umcg.nl (J.H.v.S.); a.h.brouwers@umcg.nl (A.H.B.); w.noordzij@umcg.nl (W.N.); a.t.m.willemsen@umcg.nl (A.T.M.W.); r.a.dierckx@umcg.nl (R.A.J.O.D.); a.a.lammertsma@umcg.nl (A.A.L.); a.w.j.m.glaudemans@umcg.nl (A.W.J.M.G.); c.tsoumpas@umcg.nl (C.T.); j.van.sluis@umcg.nl (J.v.S.); 2 Department of Biomedical Photonic Imaging, Faculty of Science and Technology, University of Twente, 7522 NB Enchede, The Netherlands

**Keywords:** LAFOV PET/CT, protocol optimization, parametric imaging, dual-tracer imaging

## Abstract

**Simple Summary:**

Recently: long axial field-of-view (LAFOV) positron emission tomography (PET) scanners have been introduced. Compared with conventional short axial field-of-view systems, these new scanners have a larger axial coverage and, thereby, a higher system sensitivity. This provides new opportunities for applying PET in clinical practice. Some examples are reduction of scan time duration for example in pediatric patients; reduction of the amount of radiotracer administered to the patient; longitudinal or delayed imaging using short- and long-lived radiotracers; and applications of dynamic or parametric imaging. In addition to this, new emerging techniques, such as artificial intelligence and imaging with multiple radiotracers could aid in a more general clinical application of LAFOV PET. The main objective of this review is to highlight these opportunities for oncological applications and to indicate future directions.

**Abstract:**

The latest technical development in the field of positron emission tomography/computed tomography (PET/CT) imaging has been the extension of the PET axial field-of-view. As a result of the increased number of detectors, the long axial field-of-view (LAFOV) PET systems are not only characterized by a larger anatomical coverage but also by a substantially improved sensitivity, compared with conventional short axial field-of-view PET systems. In clinical practice, this innovation has led to the following optimization: (1) improved overall image quality, (2) decreased duration of PET examinations, (3) decreased amount of radioactivity administered to the patient, or (4) a combination of any of the above. In this review, novel applications of LAFOV PET in oncology are highlighted and future directions are discussed.

## 1. Introduction

Positron emission tomography (PET) technology has significantly evolved over the last decade. The latest exciting innovation in PET/CT system design is the long axial field-of-view (LAFOV) [1]. The key feature of such a system is the substantial increase in the number of PET detectors surrounding the patient in the axial direction, allowing for a larger anatomical coverage in a single view. PET/CT systems with an axial field-of-view of 64 cm or more are generally considered to be LAFOV scanners [1].

Four LAFOV PET/CT systems have been introduced to date: the lutetium–yttrium oxyorthosilicate (LYSO) crystal-based PennPET Explorer (University of Pennsylvania), which features a 64 cm long axial FOV (planned to be extended to 140 cm) [2,3], the LYSO crystal-based uEXPLORER (United Imaging Healthcare, Shanghai, China) with 194 cm axial coverage [4], the lutetium oxoorthosilicate (LSO) crystal based Siemens Biograph Vision Quadra PET/CT (Siemens Healthineers, Erlangen, Germany) with a 106 cm long axial FOV [5], and, most recently, the Omni Legend (General Electric Healthcare, Chicago, IL, USA), which incorporates bismuth germanate (BGO) crystals and offers the possibility of a scalable axial coverage of up to 128 cm [6] (Table 1).

The two major improvements of the LAFOV PET system design are: (1) a higher photon detection efficiency because of an increased detection rate of coincidence photon pairs, and (2) a larger axial coverage, which allows for capturing all relevant organs of interest, including pathological lesions, simultaneously [7].

The higher photon detection efficiency leads to a substantially increased system sensitivity, i.e., ultra-high sensitivity LAFOV PET/CT, which allows for significant reductions in radiotracer administration to the patient, as well as for faster acquisition protocols [8,9,10,11,12,13,14]. These possibilities give rise to new applications such as ultra-low dose PET/CT imaging, longitudinal, i.e., repeat (or delayed) imaging following a single tracer injection, and multitracer PET imaging. These faster acquisitions together with potentially reduced radiation exposure open up the field of nuclear medicine and molecular imaging for a wider range of patient populations, such as intensive care and pediatric patients. In addition, increasing the frequency of follow-up scans enables better monitoring of treatment response, which could allow for a more timely change in treatment strategy, thereby resulting in more efficient and improved therapy (i.e., improved precision medicine). Furthermore, owing to the lower dose per PET examination, more scans with different tracers could be performed within one patient. As a result, drug development could be advanced by studying the biological behavior of multiple radiotracers in vivo.

This review aims to highlight present and future opportunities enabled by LAFOV PET/CT, including clinical examples illustrating these opportunities and novel applications within clinical oncology.

## 2. Scan Time Reduction

LAFOV systems offer considerable scan time reduction, whilst maintaining the image quality and administered radiotracer dose. This can result in higher patient throughput, but it can also be favorable in certain clinical settings, such as in scanning ICU patients, children, claustrophobic patients, or other patients who are unable to lie still for the duration of conventional PET/CT scans [15]. The effect of time reduction on image quality can be easily investigated by truncating the PET list mode data after the acquisition has been completed. There is no need to make complex changes or adjustments to clinical protocols, making this research topic straightforward to investigate. Therefore, many initial studies have mostly focused on the feasibility of fast or ultra-fast imaging protocols using LAFOV systems [2,9,12,16,17,18].

**Figure 1:**
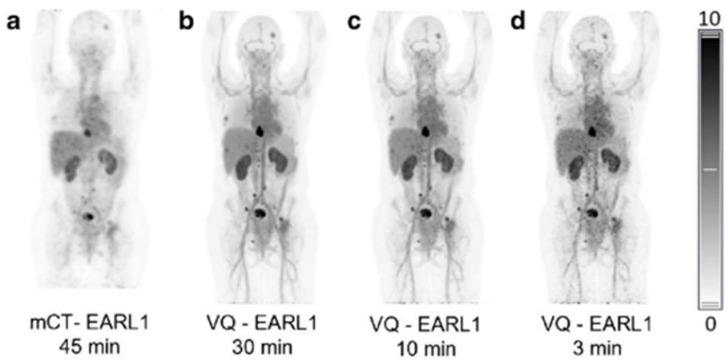
Maximum intensity projection images of a patient with metastatic HER2-positive breast cancer. Images of (**a**) a 45 min scan on a SAFOV scanner (Biograph mCT), and (**b**) 30, (**c**) 10, and (**d**) 3 min scans on a LAFOV system (Biograph Vision Quadra (VQ)). All reconstructions were according to the EARL 1 guidelines and scans were acquired 4 days after administration of 37 MBq [^89^Zr]trastuzumab. Image (partially) taken from Brouwers et al. CC BY 4.0 License: http://creativecommons.org/licenses/by/4.0/ [21].

Zirconium-89 (^89^Zr) is a long-lived PET radionuclide, primarily used in oncology for studying (tumor) target expression and whole-body biodistribution in clinical practice and research settings [19,20]. Although its half-life of 3.3 days closely matches the pharmacokinetics of monoclonal antibodies (mAbs), it also results in longer exposure to radiation, which leads to higher accumulated doses. To keep radiation exposure within acceptable limits, a limited amount of ^89^Zr can be administered. This constraint together with the low positron abundance (23%) of ^89^Zr generally results in noisy images. For this reason, conventional systems require lengthy PET acquisitions to mitigate the noise and ensure sufficient image quality, resulting in patient discomfort. Faster scans with similar or improved image quality can be achieved when highly sensitive LAFOV scanners are employed. In breast cancer patients, Brouwers and colleagues [21] demonstrated that, compared with standard protocols on a short axial field-of-view (SAFOV) system, a reduction in scan time by at least a factor of 8 could be achieved, whilst maintaining the same image quality. In this immunoPET study, HER2-positive breast cancer patients were injected with 37 MBq [^89^Zr]trastuzumab and scanned 4 days post injection on both SAFOV (Biograph mCT) and LAFOV (Biograph Vision Quadra) PET systems (Figure 1).

**Figure 2:**
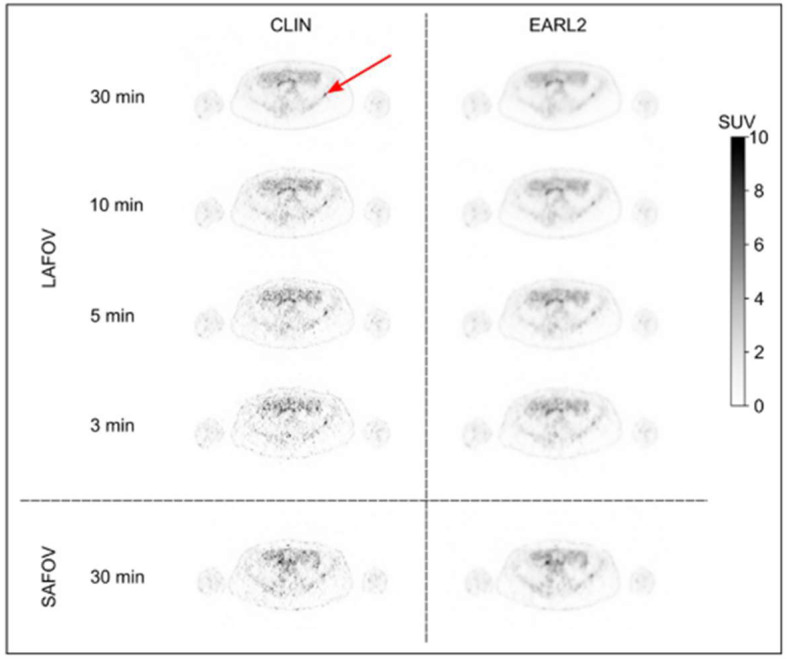
Axial images of the patient scanned on both scanners for approximately 30 min (LAFOV: Biograph Vision Quadra, SAFOV: Biograph Vision) on day 4 after injection of 37 MBq of [^89^Zr]trastuzumab, showing small metastasis in iliac bone (arrow). Images are shown for 2 reconstruction protocols and for reduced scan durations of 10, 5, and 3 min for LAFOV PET. This research was originally published in JNM. P. Mohr et al. Long Versus Short Axial Field of View Immuno-PET/CT: Semiquantitative Evaluation for (89)Zr-Trastuzumab. J Nucl Med. 2023;64(9):jnumed.123.265621. © SNMMI [22].

Mohr and colleagues demonstrated that in an immunoPET study, the semiquantitative metrics (standardized uptake values (SUVs) and coefficient of variance) of a 30 min acquisition on a standard system was comparable with a 3 min acquisition on LAFOV, which means that a reduction of a factor of 10 would provide similar quality images (Figure 2) [22].

**Figure 3:**
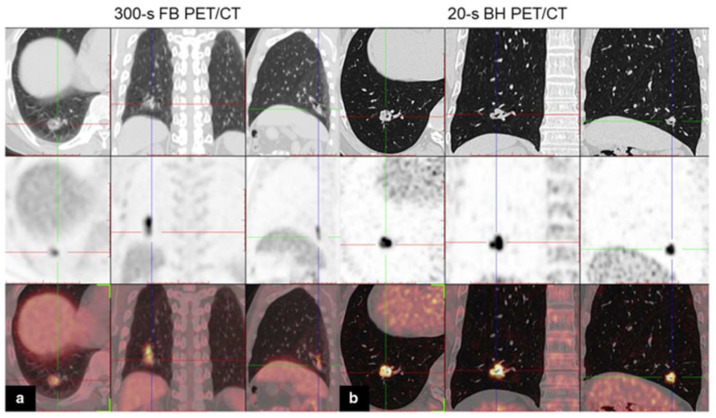
[^18^F]fluorodeoxyglucose (FDG) PET/CT images of a 59-year-old man with lung adenocarcinoma. Axial, coronal, and sagittal chest CT (**top row**), PET (**middle row**), and PET/CT (**bottom row**) images of a solitary solid lung nodule in the right lower lobe: (**a**) FB PET/CT; (**b**) BH PET/CT. Notice how the uptake area appears blurred owing to breathing artifacts in the FB PET image. Image (partially) taken from Cheng et al. CC BY 4.0 License: http://creativecommons.org/licenses/by/4.0/ [25].

In fact, the high sensitivity of LAFOV PET even allows for ultra-fast acquisitions with sufficient diagnostic image quality. Routine clinical PET examinations suffer from respiratory motion, resulting in substantial degradation in image quality due to blurring. This challenge becomes especially evident in patients with small pulmonary or liver lesions located in the proximity of the diaphragm. Early detection and characterization are impeded due to the poorer definition of these small nodules. Breath-hold (BH) studies on SAFOV PET/CT have already shown promising results in mitigating such effects [23,24]. Nevertheless, the high sensitivity of LAFOV PET/CT makes breath-hold acquisitions realistic. For example, Cheng et al. showed that with 20 s BH FDG PET/CT acquisitions, the blurring of pulmonary adenocarcinoma was reduced, resulting in improved lesion detectability and quantitative parameters, such as the standardized uptake values (SUVs) and tumor-to-background ratio (TBR) [25]. In this retrospective study, 47 patients underwent a 20 s breath-hold acquisition in addition to a 300 s free-breathing scan. Figure 3 shows the improved lesion definition of a solitary lung nodule in a 59-year-old patient with lung adenocarcinoma.

**Figure 4:**
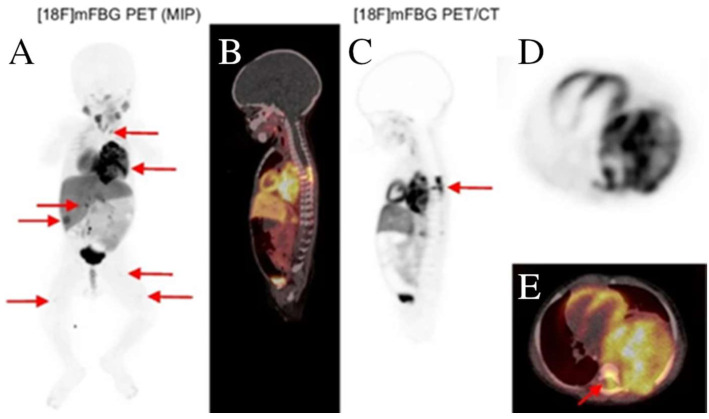
The short-duration [^18^F]meta-fluorobenzylguanidine ([^18^F]mFBG) PET scan of a 9-month-old pediatric patient revealed several lesions (red arrows). (**A**) Maximum intensity projection images of a 2 min reconstruction. (**B**,**C**) Sagittal views of co-registered PET/CT and PET-only images. (**D**,**E**) Transaxial views of PET-only and co-registered PET/CT images. Image (partially) taken and modified from Borgwardt et al. CC BY 4.0 License: http://creativecommons.org/licenses/by/4.0/ [29].

Furthermore, one of the main challenges in medical imaging of children is gross body motion [26]. Motion artifacts in PET can result in, among others, blurring, reduced contrast, and increased image noise [27]. General anesthesia could reduce gross body or voluntary motion, but preclinical studies have suggested that anesthetic agents may be neurotoxic for a developing brain [28]. The challenge of motion artifacts in PET could be met by imaging pediatric patients using LAFOV systems. The increased sensitivity not only improves overall image quality but also reduces motion artifacts simply because of the faster acquisitions. Borgwardt and colleagues [29] demonstrated that a fast LAFOV PET/CT scan could be performed in a 9-month-old patient without the need for general anesthesia. The scan accurately identified the extension of a neuroblastoma, which is significant for staging and treatment purposes (Figure 4). The list mode scan of 10 min duration was acquired 62 min after injecting a dose of 30 MBq (3 MBq/kg) F-18 labeled meta-fluorobenzylguanidine ([^18^F]mFBG). It turned out that only 2 min were needed for reconstruction, resulting in a clinically valuable image without any motion artifacts.

## 3. Dose Reduction

In addition to scan time reduction, the amount of radioactivity administered to the patient could also be reduced. The radiation exposure caused by PET/CT scans is composed of two sources: the PET tracer and the X-ray CT. In 2005, Brix and colleagues reported that the average effective dose received by patients in four German hospitals during a whole-body [^18^F]fluorodeoxyglucose (FDG) PET/CT scan was approximately 25 mSv, consisting of 5.7–7.0 mSv for the PET scan and 14.1–18.6 mSv for the whole-body CT scan [30,31]. According to a more recent nationwide survey conducted in Korea [32], involving a total of 105 PET/CT scanners across 73 institutions, the average effective dose from FDG and CT was found to be 5.89 ± 1.46 mSv and 6.26 ± 3.06 mSv, respectively. The study also revealed that the radiation dose from FDG PET/CT was significantly reduced when using newer scanners [32]. The injected activity went from 6.10 ± 1.19 MBq/kg for older scanners (installed before 2005) to 4.60 ± 0.85 MBq/kg for newer scanners (installed after 2011). The volume computed tomography dose index (CTDI_vol_) went from 6.04 ± 2.58 mGy for older scanners to 3.95 ± 1.97 mGy for the newer scanners.

Radioactive tracers that are used for PET/CT scanning could lead to radiation-related risks, which is a concern for the more radiosensitive patient population, such as children and pregnant women, due to lifetime susceptibility to cancer [15]. This risk is especially a concern when multiple examinations are required, which is common for the evaluation of treatment response. Accordingly, in response to this concern, legal frameworks, such as the EURATOM 2013/59 Directive and the Netherlands Commission on Radiation Dosimetry (NCS), have been established to regulate radiation exposure to patients [12,33,34]. Although reducing the amount of radioactive tracer can decrease radiation exposure, it also leads to increased noise, more artifacts, and a lack of contrast, thereby potentially affecting diagnosis. PET image quality relies on the amount of injected radiotracer and the duration of the scan, i.e., the number of counts acquired, which, in turn, determines the noise properties of reconstructed PET images. The trade-off between radiation exposure and image quality has gained research interest worldwide [35].

Previous investigations using the recently introduced LAFOV systems have demonstrated improved photon detection efficiency as a result of the increased number of detectors, making low-dose examination protocols feasible. In fact, a reduction in administered dose by a factor of up to six is possible [4,5,35].

**Figure 5:**
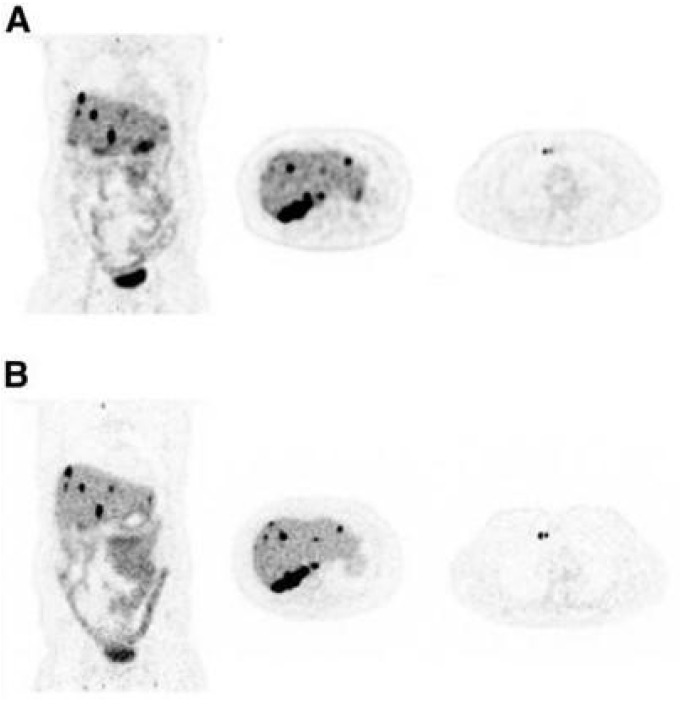
(**A**) The [^68^Ga]DOTATATE PET images using the clinical scan (standard of care) show coronal and transverse views of a subject with a metastatic neuroendocrine tumor. (**B**) Coronal and transverse images of the same subject were taken on the PennPET scanner, acquired 3.5 h after injection, with a 20 min scanning duration. Image (partially) taken from Pantel et al. CC BY 4.0 License: http://creativecommons.org/licenses/by/4.0/ [2].

Pantel et al. [2] conducted a study using the PennPET Explorer system showing diagnostic image quality comparable to that of a conventional clinical scan using only a factor of five times less administered activity. To evaluate images obtained with a low amount of radioactivity, delayed images were acquired using [^68^Ga]DOTATATE for a subject with metastatic neuroendocrine cancer (Figure 5). At the time of acquisition, 3.5 h after injection, the amount of activity had decayed to one fifth of the original administrated amount effectively corresponding to an injected activity of approximately 30 MBq. Despite this difference in activity, the resulting images have comparable image quality.

**Figure 6:**
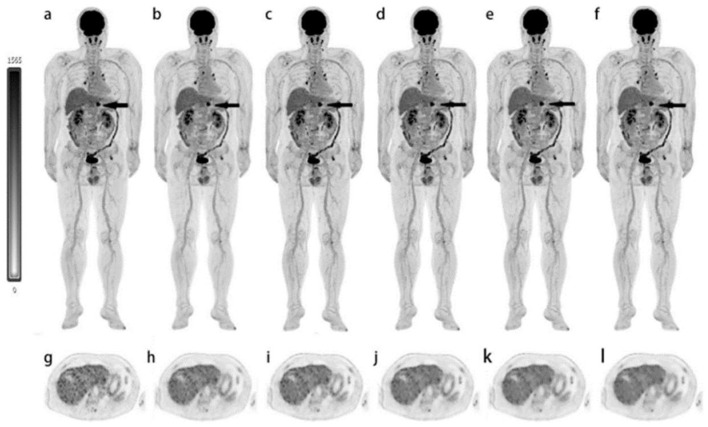
MIP and trans axial view reconstructed PET images of a 62-year-old man with surgically confirmed pancreatic cancer. Different reconstruction techniques were used (OSEM3 (**a**,**g**), OSEM2 (**b**,**h**), HYPER0.3 (**c**,**i**), HYPER0.4 (**d**,**j**), HYPER0.5 (**e**,**k**), and HYPER0.6 (**f**,**l**)). The images show increased FDG uptake in the pancreas. The overall image scores of 2, 3, 3, 3, 4, and 4 were given to the 6 groups, respectively (Scores of 5, 4, or 3 were considered to meet the diagnostic requirements, and scores of 2 or 1 indicated sub-optimal or non-diagnostic image quality). Image (partially) taken from Sui et al. CC BY 4.0 License: http://creativecommons.org/licenses/by/4.0/ [35].

In another study, Sui et al. [35] demonstrated that LAFOV PET/CT with an ultra-low FDG dose (0.37 MBq/kg) resulted in satisfactory image quality and diagnostic performance. List mode PET data were acquired for 15 min using the uEXPLORER PET/CT system. In patients with a body mass index (BMI) below 30, OSEM reconstructions with three iterations and 20 subsets provided images of sufficient quality for diagnostic reading purposes. However, for patients with a BMI equal to or higher than 30, the image quality was not sufficient. For this patient population, the HYPER iterative algorithm for PET image reconstruction was recommended, which is a new Bayesian penalized likelihood reconstruction algorithm to ensure consistent visual image quality and accurate quantitative assessment (Figure 6).

Pediatric examinations benefit from low-dose imaging on LAFOV as well, especially to reduce the risk of future carcinogenesis. For example, Zhao et al. [36] showed the potential of LAFOV PET/CT in reducing the administered dose, whilst maintaining image quality and diagnostic performance for micro-lesions in pediatric patients. A total of 33 pediatric patients underwent LAFOV PET/CT scans using the uEXPLORER PET/CT with an administered FDG dose of 3.7 MBq/kg and an acquisition time of 600 s. Subsequently, additional low-dose images were obtained by only using a part of the rebinned list mode PET data, resulting in additional PET images with reduced count density. This study demonstrated that image quality could be maintained with a reduction in administrated activity by a factor of 10 (0.37 MBq/kg).

**Figure 7:**
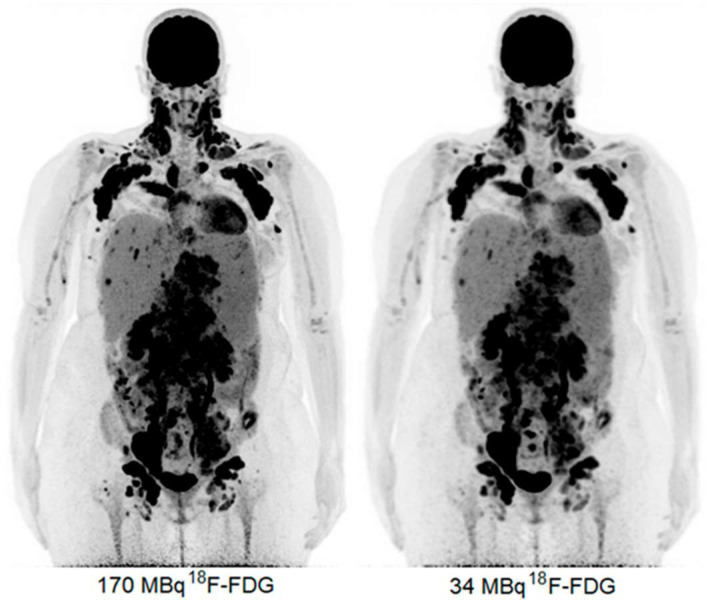
Maximum intensity projection (MIP) PET images acquired via a LAFOV Biograph Vision Quadra PET/CT scanner in a patient at 19 weeks of pregnancy with 170 MBq FDG (**left**) and reconstructed with 34 MBq FDG (**right**). Image (partially) taken from van Sluis et al. CC BY 4.0 License: http://creativecommons.org/licenses/by/4.0/ [39].

PET/CT scanning during pregnancy presents the ultimate hurdle that necessitates expert interdisciplinary coordination. Determining whether to proceed with a nuclear medicine examination of a pregnant woman requires a thorough evaluation of the clinical advantages for the mother against the potential risks of radiation exposure to the fetus [37,38]. Figure 7 [39] shows a patient at 19 weeks of pregnancy diagnosed with follicular lymphoma and scanned using LAFOV FDG PET for staging and therapy choice. PET list mode data were rebinned and only a part corresponding to a factor of 10 reduction in administered activity was used (equivalent to an injected activity of 34 MBq, e.g., 0.3 MBq/kg). The resulting image showed acceptable diagnostic quality visually, clearly highlighting the areas of lymphadenopathy. This indicates that the average fetal radiation exposure can be reduced from 3.7 to 0.7 mGy [39].

As mentioned above, the radiation dose in PET/CT imaging results from a combination of administered activity and the radiation dose originating from the CT scan. Previously, it has been reported that, even with existing dose reduction techniques and protocol optimization for the CT component [13,40,41], a low-dose non-contrast enhanced CT scan for attenuation correction can still expose patients to a radiation dose of 1–3 mSv. An interesting method to reduce the CT radiation dose involves the use of a removable tin filter, which has the potential to reduce the radiation dose by 90% in a whole-body diagnostic CT scan without affecting the quantification or quality of PET images [42]. The LAFOV PET/CT Quadra scanner is equipped with a tin filter, which serves as a high-pass filter. Lower-energy photons, which do not add to the image quality, are removed from the beam, thereby decreasing the dose delivered to the patient.

## 4. Longitudinal and Delayed Imaging

The high sensitivity of LAFOV PET/CT also allows for PET scans at later time points than commonly used for conventional SAFOV scanners. Typically, a PET tracer shows high activity in the blood pool immediately after injection and subsequently accumulates in the target regions over time. FDG, for example, shows increased uptake in tumor sites up to 8 h after injection [43]. The specific signal of an irreversible tracer like FDG in lesions often increases in delayed imaging, whereas the nonspecific signal decreases [44], leading to a better target-to-background (TBR) ratio. However, due to the decay of the radiotracer, the later the imaging is performed, the more radioactivity has decayed, resulting in lower count rates and noisier images. The higher sensitivity of LAFOV PET/CT scanners means that PET images can be obtained with a similar signal-to-noise ratio as SAFOV images, but at scan times that are up to four to five half-lives later [2,45]. For the commonly used shorter-lived tracers labeled with ^18^F (T_1/2_ = 109.8 min), ^68^Ga (T_1/2_ = 67.7 min), or ^11^C (T_1/2_ = 20.4 min), this corresponds to about 9 h, 6 h, and 1.5 h post injection, respectively.

**Figure 8:**
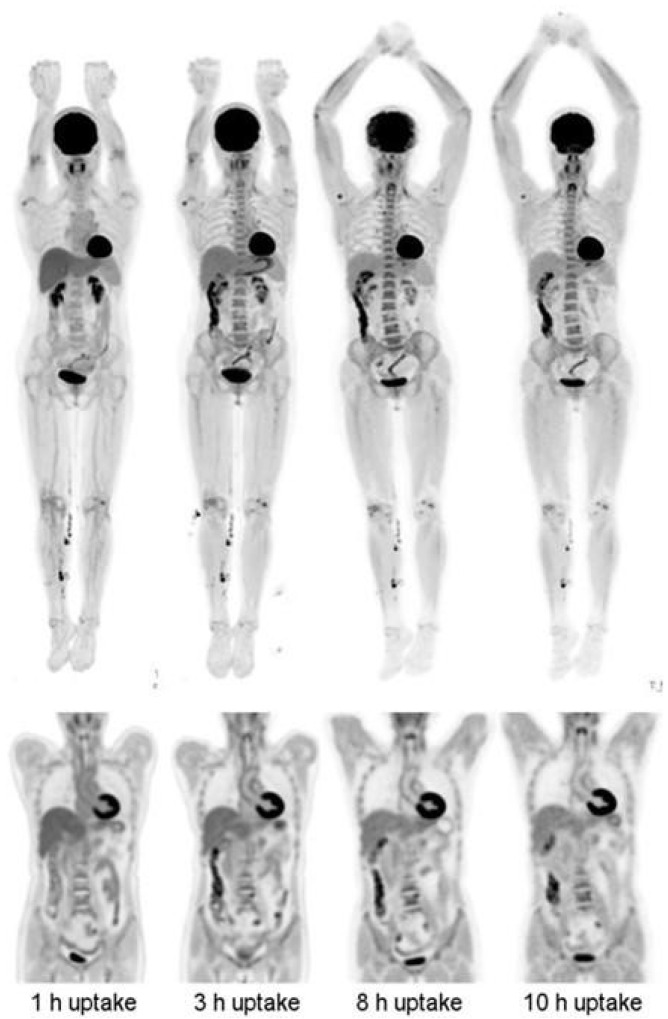
(**Left**-to-**right**) Images from 14 min scans performed at 1, 3, 8, and 10 h after injection of 256 MBq FDG. (**Top row**) MIP images. (**Bottom row**) Coronal views of thorax and abdomen. This research was originally published in JNM. R.D. Badawi et al. First Human Imaging Studies with the EXPLORER Total-Body PET Scanner. J Nucl Med. 2019;60(3):299–303. © SNMMI [46].

**Figure 9:**
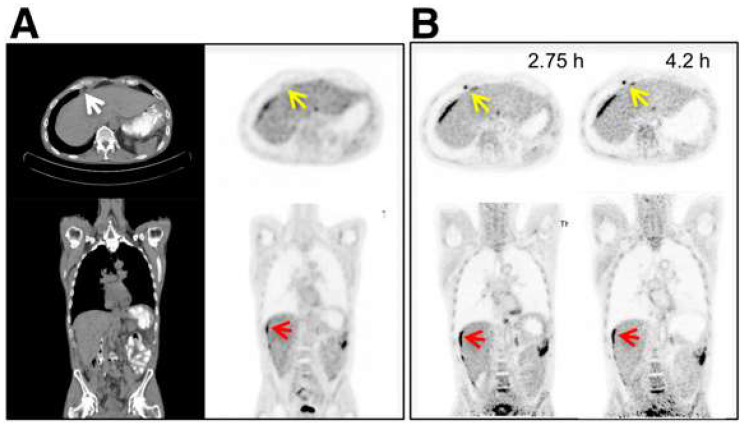
(**A**) Clinical FDG PET/CT images (transverse and coronal) from subject 5, with metastatic colon cancer, acquired with standard clinical protocol (SAFOV). (**B**) PennPET (LAFOV) image acquired 2.75 and 4.2 h after injection (10 min scans). Matched coronal and transverse slices are shown. Red arrows denote perihepatic disease; yellow arrows denote epiphrenic lymph node. Image (partially) taken from Pantel et al. CC BY 4.0 License: http://creativecommons.org/licenses/by/4.0/ [2].

**Figure 10:**
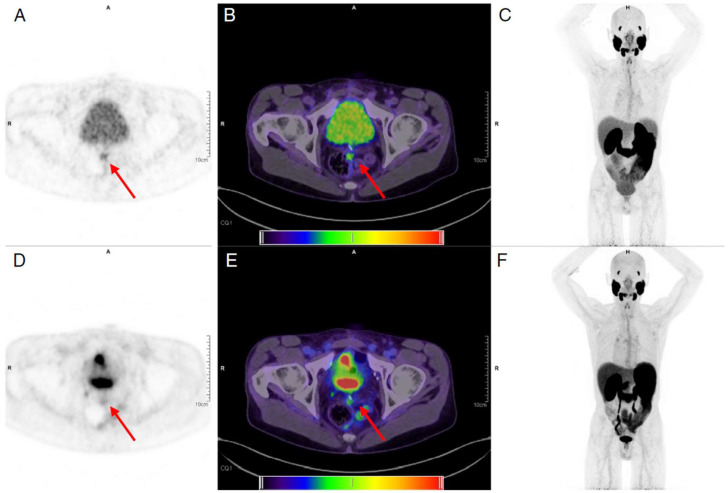
Illustrative [^68^Ga]Ga-PSMA-11 images for an example patient. Shown are images acquired at 4 h p.i. with a 16 min total acquisition time (top row, tiles (**A**–**C**)) and 1 h p.i. images with 6 min total acquisition time (bottom row, tiles (**D**–**F**)). Visual inspection of the two maximal intensity projections (**C**,**F**) demonstrates that only a modest reduction in image quality is seen at late imaging. The locally recurrent lesion (shown by red arrows) at the left mesorectal fascia is faintly visible at 1 h (PET (**D**) and fusion PET and CT, (**E**)) but more clearly discerned at 4 h (PET (**A**) and fusion PET and CT (**B**)). For reference, the PET window was set to 0 and 6 SUV to best display the image (partially) taken from Alberts et al. CC BY 4.0 License: http://creativecommons.org/licenses/by/4.0/ [47].

In Figure 8, examples are shown of a 14 min FDG scan (injected activity: 256 MBq) in a healthy volunteer acquired up to 10 h after injection on the uEXPLORER [46]. At the later time points, there is less activity in the blood pool compared with the 1 h time point. Another example of delayed FDG imaging is shown in Figure 9. In this study by Pantel and colleagues [2], the perihepatic disease is more clearly visible on the two delayed time points (2.75 and 4.2 h after injection) compared with the standard PET study, in part because of clearance of FDG from the non-diseased adjacent liver. In addition, the PennPET scan reveals an avid lymph node that was not identified on the standard scan. Furthermore, Alberts et al. published another delayed [^68^Ga]PSMA study that is shown in Figure 10 [47]. Imaging was performed for 6 min at 1 h, as well as 16 min at 4 h after injection on a LAFOV Quadra PET/CT scanner. Both images resulted in visually similar image quality but with improved lesion discernibility. A metastatic lymph node is clearly visible on the delayed image, whereas it is only faintly visible on the baseline scan.

**Figure 11:**
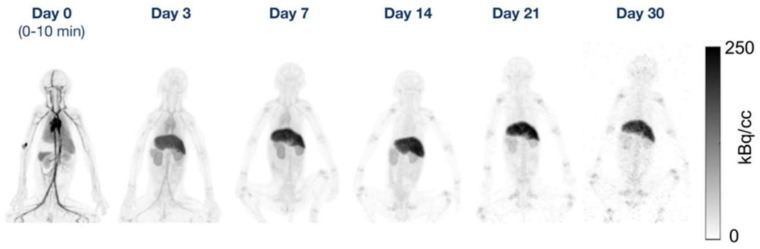
Maximum intensity projection PET images at each time point for 1 rhesus monkey in the 89Zr-DFO-squaramide–anti-gD group. This research was originally published in JNM. E. Berg et al. Total-Body PET and Highly Stable Chelators Together Enable Meaningful 89Zr-Antibody PET Studies up to 30 Days After Injection. J Nucl Med. 2020;61(3):453–460. © SNMMI [45].

^89^Zr immunoPET imaging is an application that inherently requires late images due to the slow kinetics of mAbs. For a given new ^89^Zr-labelled tracer, the optimal day for PET imaging is determined and usually ranges from 4 days to 1 week for a full mAb [48,49,50]. Although later time points are expected to provide optimal TBR, the associated increase in image noise would require unacceptably long acquisitions of up to 2 h on current SAFOV systems, thus in practice often a compromise for an earlier scanning time is made. At the time of writing, there have been no reports yet on human immunoPET images acquired at substantially delayed scan times. However, Berg et al. [45] have reported on a study using the mini-EXPLORER (45 cm FOV) to image ^89^Zr-labelled antibodies up to 30 days after injection in nonhuman primates (Figure 11).

Longitudinal imaging refers to scanning at multiple time points, which can be applied to short-lived or long-lived tracers. For example, it has been shown that dual-time-point imaging using FDG can help to differentiate malignant from inflammatory processes [51]. In malignant tissue, FDG uptake keeps increasing over time, whereas in inflamed or infected tissues, the FDG uptake may drop following an increase [52]. Dual-time-point imaging has also shown to be promising in distinguishing between tumor activity and inflammation after anti-cancer therapy, such as radiotherapy and/or chemotherapy [53].

## 5. Parametric Imaging

Simple standardized uptake value (SUV) measurements allow clinicians to quantify the intensity of radiotracer uptake in a tumor, which usually reflects its metabolic activity and thereby its aggressiveness. SUV provides insights into prognosis and the assessment of the response of a patient to treatment. Despite being widely used in both clinical practice and cancer research studies, the use of SUV is not unanimously accepted, as it is subject to multiple sources of variability [54,55]. These are a consequence of several factors including (1) factors external to the patient, such as the use of different scanners and/or imaging protocols and partial volume effects; (2) factors intrinsic to the patient, such as patient weight, liver and kidney function, and plasma glucose levels; and (3) other biological factors such as blood flow and tracer clearance [56]. On the other hand, rate constants derived from pharmacokinetic modeling can give a good approximation of the underlying biological processes by accounting for factors such as tracer delivery, tissue perfusion, and transport rates (e.g., tracer uptake and clearance over time). Fully quantitative methods include both tissue compartment models and graphical analyses and are used to estimate specific parameters such as blood flow, perfusion, receptor density, and tissue metabolism. However, several hurdles related to pharmacokinetic modeling have hindered its implementation in clinical practice. These hurdles include (1) the need for long scan durations of 60–90 min, apart from some potentially fast radiotracers; (2) the reduced FOV of standard PET scanners (up to 26 cm), which only allows information to be dynamically acquired from one region of the body simultaneously; and (3) the need for an input function either from an artery (invasively or non-invasively) or from a blood pool reference region. LAFOV systems with an extended FOV have caused a paradigm shift in the field of fully quantitative imaging, making it feasible to overcome some of the previously mentioned hurdles and facilitate its application in clinical practice.

### 5.1. Image-Derived Input Function

**Figure 12:**
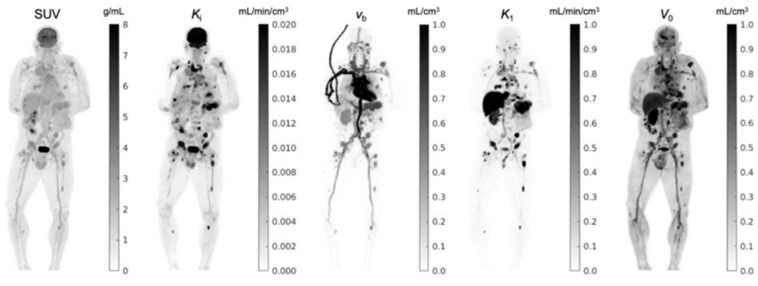
Comparison of standard SUV image with parametric images of FDG influx rate K_i_, fractional blood volume v_b_, FDG delivery rate K_1_, and volume of distribution V_0_ of a cancer patient. Shown are maximum intensity projection maps. This research was originally published in JNM. G. Wang et al. Total-Body PET Multiparametric Imaging of Cancer Using a Voxelwise Strategy of Compartmental Modeling. J Nucl Med. 2022;63:1274–1281. © SNMMI [61].

LAFOV PET systems have revolutionized non-invasive tumor quantification by enabling the acquisition of simultaneous dynamic images over the entire body. These dynamic images encompass large blood pools that serve as a non-invasive and practical alternative to the conventional arterial input function [57] when metabolic corrections can be neglected. Several studies have validated the efficiency of LAFOV PET for the quantification of kinetic FDG parameters with an image-derived input function (IDIF) for both healthy tissue [58,59,60] as well as tumors [61,62]. Together with the increased axial coverage, the increased sensitivity and temporal resolution of whole-body PET scans enable the obtainment of parametric images, which have been shown to improve tumor contrast against the background, e.g., the liver, compared with SUV images (Figure 12) [61,62]. Furthermore, in addition to K_i_, kinetic modeling allows for a deeper knowledge of tumor biology by providing information on K_1_ (tracer delivery rate), V_T_ (volume of distribution), and BP_ND_ (non-displaceable binding potential). Apart from FDG, regional kinetic modeling and parametric imaging have also been applied to dynamic [^68^Ga]FAPI-04 scans in patients with pancreatic and gastric cancer, [^15^O]H_2_O scans for perfusion studies, and [^18^F]FET and [^11^C]methionine in patients with brain tumors [63,64,65].

### 5.2. Shortened Scan Protocols

The dynamic studies mentioned above may require a prolonged dynamic PET acquisition (60–90 min), which is not only uncomfortable for the patient but also impractical in a clinical context. With the introduction of LAFOV PET/CT systems, several alternatives have been proposed for deriving the kinetic parameters of FDG scans by using shortened dynamic imaging protocols in conjunction with Patlak analysis. Shortened scan protocols are specifically relevant for LAFOV scanners because of the larger axial coverage in a single-bed position, enabling the inclusion of large blood pools to extract an IDIF, as well as the superior system sensitivity associated with these scanners, which significantly decreases image noise. There are four types of shortened scan protocols, the details of which are described in the following sections.

#### 5.2.1. Late-Time Scanning

In late-time scanning protocols, imaging data are acquired only during the later stages after tracer injection. This means that information regarding early frames is missing and, therefore, alternative approaches are needed to recover this information. One option is to use population-based input functions (PIFs), which involve generating a statistical model that estimates the plasma input function for a specific tracer in a population of subjects. To perform Patlak analysis with late-time scanning protocols, a PIF can be used to scale the IDIF extracted during the last time frames and Patlak K_i_ values can be generated using the scaled curve [66,67]. One caveat in this approach is that the assumption has to be made that the shape of the input curve is the same for every patient. And, even if this approach may be affected by some potential inaccuracies, the fact is that kinetic parameters can be estimated from a reasonably short scan (i.e., around 5 min for FDG). Alternatively, it has also been shown that a Patlak slope obtained from data of late frames can be normalized using a global scaling factor, thus eliminating the need for a PIF [68]. Potentially, there are major advantages to implementing these late scanning protocols. First, the resulting parametric images may, just like parametric images from full scanning protocols, show better TBR than corresponding SUV images. Second, the scanning procedure could possibly end earlier than when a static scan is obtained, as the latter requires sufficient uptake time.

#### 5.2.2. Dual-Window Imaging

**Figure 13:**
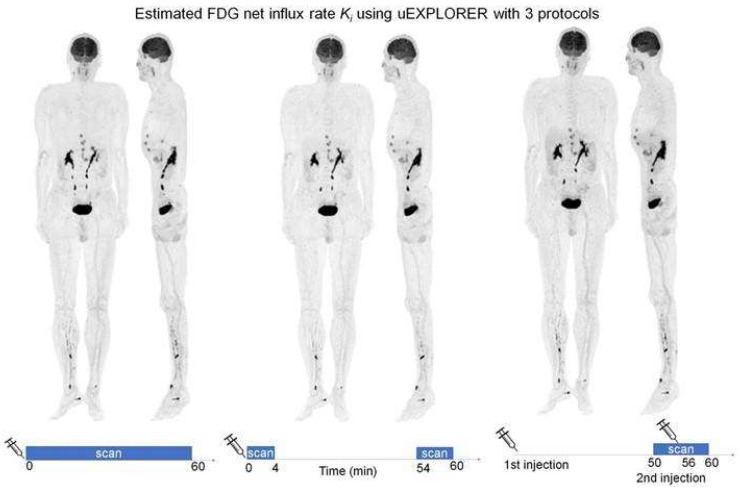
Maximum intensity projection PET images of FDG influx rate K_i_ from three different protocols: standard 60 min dynamic scan (**left**), dual-scan imaging (**middle**), and second injection scan (**right**). This research was originally published in JNM. Y. Wu et al. Whole-Body Parametric Imaging of (18)F-FDG PET Using uEXPLORER with Reduced Scanning Time. J Nucl Med. 2022;63:622–628. © SNMMI [16].

In a dual-window imaging protocol, two separate dynamic scans, both with short duration, are acquired. The first scan is obtained immediately after tracer injection (i.e., first minutes), while the second scan is acquired during the final minutes of a standard scanning protocol (e.g., last ten minutes). In FDG scans, the full input function needed for calculating Patlak images can then be obtained using a hybrid approach that adjusts a PIF so that it coincides with an IDIF extracted from both early and late scans [16] (Figure 13).

#### 5.2.3. Second Injection

In a second injection protocol, a single scan is acquired during the last 10 min of a standard 60 min dynamic scan, with a second injection being performed around halfway through the acquisition. To obtain the full input function, the first and second half of the scan are used in the reverse order. The contribution of the second injection is estimated and treated as the early phase IF, whereas the part before the injection (i.e., 50–55 min) is treated as the final part of the scan (e.g., the last 5 min). The missing part in the middle is obtained in the same way as in the dual-scan imaging protocol [16] (Figure 13).

#### 5.2.4. Early Time Scanning

With this protocol, a patient is scanned only during the first 30 min post injection, and the image is reconstructed using very short timeframes (between 1 and 8 s). K_i_ parametric Patlak images are then obtained by applying a denoising method to the calculated voxel data before linear fitting with the standard Patlak model equation [69]. Although this protocol also has been used in standard axial field-of-view systems, shorter scan durations are too noisy for quantification [70]. Nonetheless, shorter scan durations with adequate image quality are possible using LAFOV, as a result of the higher sensitivity [8].

**Figure 14:**
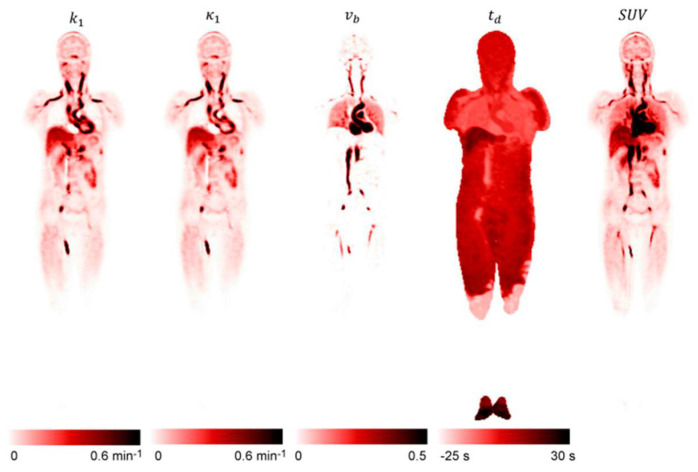
The coronal plane of k_1_ (delivery rate), v_b_ (blood fraction in the tissue), and t_d_ (time delay) parametric images were reconstructed using only the first 90 s post FDG injection. The SUV image acquired from the same period is also shown for comparison. This research was originally published in JNM. T. Feng et al. Total-Body Quantitative Parametric Imaging of Early Kinetics of (18)F-FDG. J Nucl Med. 2021;62:738–44. © SNMMI [71].

Finally, T. Feng et al. [71] have shown that it is possible to obtain FDG whole-body parametric images of K_1_ using only the first 90 s post injection (Figure 14). Although this specific study was not performed in oncological patients, in general, the K_1_ could provide valuable information regarding tumor blood flow and metabolism [64,72], tumor differentiation [73], tumor gene expression [74], and response to therapy [72,75].

## 6. Dual Tracer

Compared with single-tracer examinations, dual-tracer studies provide valuable additional information, as two radiotracers are used to comprehensively characterize biological processes. For example, the combination of ^18^F and ^11^C tracers, as well as ^18^F and ^68^Ga tracers, have emerged as particularly valuable in providing insights into different disease characteristics. The pair of ^18^F and ^11^C enables the assessment of neurochemical changes and glucose metabolism in neurodegenerative disorders such as Parkinson’s disease [76], while also offering valuable information on metabolic alterations associated with de novo lipogenesis in hepatocellular carcinoma studies [77]. On the other hand, the combination of ^18^F and ^68^Ga allows for the visualization of somatostatin receptor expression ([^68^Ga]DOTATOC) and assessment of the dopaminergic system ([^18^F]FDOPA), which can be used to determine the characteristics of pulmonary carcinoids and guide the selection of the most suitable therapy [78]. However, dual tracer studies are currently performed by administering and imaging the two tracers sequentially. As a result, patients receive a radiotracer injection and are scanned twice, including the double CT scan. Conversely, as a result of the high sensitivity of LAFOV systems, substantial dose reduction can be achieved compared with conventional systems. This opens up the possibility of administering two tracers simultaneously. The main advantage of this technique is that the patient undergoes only a single scan, increasing patient comfort and making co-registration of multiple medical images superfluous. The latter is especially important when patients are under unstable conditions, i.e., following a systemic or local intervention [79].

Despite the low doses, the separation of the signal of two tracers becomes possible using temporal information (i.e., radionuclide decay and tracer kinetics), due to the high sensitivity of LAFOV. The combination of two tracers in a single scan within clinically acceptable acquisition times and cumulative patient radiation exposure limits can offer a substantial leap in the use of PET imaging, especially in situations where molecular changes alter very rapidly, like in drastic cancer treatments [79]. A recent study by Liu et al. proposed a new scanning protocol for FDG and [^68^Ga]Ga-DOTA-FAPI-04 with shorter scan duration and less administered radioactivity [80]. In this protocol, a low-dose CT was first performed for attenuation correction followed by a 10 min low-dose static FDG (0.37 MBq/kg, one tenth of the standard amount of activity) scan, which was immediately followed by an additional 60 min dynamic scan after injection of a low dose of [^68^Ga]Ga-DOTA-FAPI-04 (0.925 MBq/kg, half of the standard amount of activity). The results showed improved image quality and lesion detection including lesion number, extent of the disease, and contrast, whilst radiation exposure was reported to be comparable to a single standard whole-body FDG PET/CT acquisition [80].

## 7. Drawbacks

The opportunities for transforming the landscape of cancer response monitoring and response prediction have expanded substantially with the advent of LAFOV PET technologies. At present, the main limiting factors are not only the costs of purchasing, installing, and maintaining such a scanner, but also updating the patient setup in order to be able to process a higher throughput of patients. Although it is difficult to specify the exact increase in costs compared with SAFOV systems, it is generally proportional to the axial length of the system (excluding CT costs). Vandenberghe and colleagues [7] estimated the costs of LAFOV systems of different lengths based on the component costs of LAFOV systems. Compared with an SAFOV of 20 cm, the costs of 1, 1.4, and 2 m LAFOV scanners were estimated to be 4, 5.5, and 7.7 times higher, respectively [7]. In addition to the high costs of acquiring and maintaining such tomographs, centers are required to have adequate space to harbor LAFOV systems, taking into consideration that the design of many rooms is only suitable for traditional scanners. Moreover, waiting rooms, rooms for preparation, and relaxing rooms need to be adjusted to accommodate a higher number of patients. This may lead to enormous disparities, not only between countries and healthcare systems that can afford such scanners but also between patients of the same department where decisions need to be made about who will be scanned on a SAFOV and who will be scanned on an LAFOV system. The scientific community needs to develop much lower costs, more affordable PET scanners [81,82,83], or else provide compelling evidence that would justify the installation of a large number of LAFOV scanners around the world, e.g., by showing that many more scans per hour can be performed or by showing substantially improved performance in detection disease or, more likely, in selecting the most appropriate treatment. Conversely, due to the improved quality of data acquired on LAFOV systems, the storage and processing of such data can pose a challenge and will require careful consideration regarding storage and IT infrastructure.

## 8. Future Perspectives

The potential of LAFOV systems to decrease the injected radioactivity could potentially enable the involvement of healthy volunteers in clinical drug development trials or for screening purposes in the oncological field. In addition, lowering the amount of radioactivity of long-lived tracers, such as labeled monoclonal antibodies (mAbs) in immunoPET, could allow for drug development and prediction of therapy response in other indications than as a last resort in oncology (i.e., infection and inflammation).

Artificial intelligence has had a substantial global impact across all fields, and nuclear medicine is no exception. Numerous machine or deep learning techniques have been developed to reduce noise, and nonlinear transformations have been studied to predict standard doses based on low-dose images [84,85,86,87]. In addition, manufacturers have made attempts to improve PET image quality by developing AI filters that can perform Time-of-Flight (ToF) modeling, even for non-ToF systems [88]. Currently, studies are exploring innovative methods for PET attenuation correction without the need for a CT scan. Some of these approaches apply artificial intelligence (AI)-based strategies [89] or the generation of attenuation maps from background radiation events that naturally occur in LSO [90]. Nevertheless, even the low-dose CT scans offer crucial anatomical (e.g., localization) details. Recent advancements in CT scanners have introduced dose optimization parameters, allowing for further reduction in CT radiation dose [91,92].

Furthermore, longitudinal imaging of long-lived radiotracers can provide kinetic information, similar to a dynamic scan, but with fewer sample points. The high sensitivity of LAFOV PET/CT could enable longitudinal imaging for ^89^Zr immunoPET, thereby leading to more data required to fit tracer kinetic models. Patlak modeling of multiple time-point immunoPET data has already been evaluated with conventional scanners [93,94] but will benefit from the increased sensitivity of LAFOV PET systems, allowing for lower noise levels, further delayed time points, and shorter scan durations.

Additionally, LAFOV PET scanners have opened a door for performing non-invasive kinetic modeling across a broader range of tracers. Until now, full non-invasive quantification has been limited to tracers without metabolites (e.g., FDG and ^15^O), which only require arterial whole blood concentrations for determining the arterial input function. By enabling the simultaneous dynamic analysis of multiple organs with different kinetics, it might be possible to use techniques such as simultaneous fitting to derive a metabolite-corrected input function non-invasively [95,96].

For future application of the dual-tracer approach for tracers other than ^18^F, ^11^C, and ^68^Ga, further analysis for each new tracer is required. In addition, simultaneous imaging of multiple tracers could promote patient comfort and reduce the logistical challenges of a busy PET clinic. Based on the physical principle underlying the differentiation of two or more simultaneously injected tracers, a formidable challenge lies ahead. The high sensitivity and large anatomical coverage of LAFOV PET/CT open up new opportunities for the separation of dual tracers as individual signals by employing dynamic protocols to distinguish each tracer’s kinetic behavior, or alternatively, by utilizing prompt gammas and triplet imaging [13,97,98].

## 9. Conclusions

With the introduction of LAFOV, PET acquisitions can now be performed considerably faster, with substantially lower doses and/or with significantly increased image quality, enabling novel applications of this modality. Moreover, the extended axial field-of-view of such tomographs allows for capturing the most important organs in one-bed position, enabling faster dynamic imaging acquisitions and/or improved image quality compared with conventional systems. In the near future, artificial intelligence tools, which are currently under development, could be implemented in standard clinical practice in order to promote additional optimization in acquisition time and/or dose reduction. Moreover, the high sensitivity and extended axial coverage of LAFOV could facilitate the application of dual-tracer imaging, promoting a more comprehensive understanding of essential biological processes. Alternatively, future implementations could be the translation to non-oncological applications as a standard of clinical care, e.g., immunoPET for drug development in inflammatory and infectious diseases.

## Figures and Tables

**Table 1:** Characteristics of current long axial field-of-view PET/CT scanners.

Characteristics\System	PennPET Explorer	uExplorer	Biograph Vision Quadra	Omni Legend
Manufacturer	University of Pennsylvania, KAGE Medical, and Philips	UC Davis and United Imaging Healthcare	Siemens Healthineers	General Electric Healthcare
Axial field-of-view (cm)	140 ^1^	194	106	128 ^1^
Scintillator	LYSO ^2^	LYSO ^2^	LSO ^2^	BGO ^2^
Sensitivity (kcps/MBq)	55	174	174	46

^1^ The current PennPET Explorer has an axial field-of-view of 64 cm but is planned to be extended to 140 cm. The Omni Legend has a scalable design and can be extended up to 128 cm. ^2^ LYSO: lutetium–yttrium oxyorthosilicate, LSO: lutetium oxoorthosilicate, and BGO: bismuth germanate.
